# A Low-Sidelobe Fully Metallic Ridge Gap Waveguide Antenna Array for W-Band Applications †

**DOI:** 10.3390/s26020602

**Published:** 2026-01-15

**Authors:** Huixia Jiang, Lili Sheng, Pengsheng Nie, Yu Feng, Jinfang Wen, Jianbo Ji, Weiping Cao

**Affiliations:** 1 Guangxi Key Laboratory of Wireless Wideband Communication and Signal Processing, School of Information and Communication, Guilin University of Electronic Technology, Guilin 541004, China; 2 School of Artificial Intelligence, Guilin University of Aerospace Technology, Guilin 541004, China

**Keywords:** ridge gap waveguide, low-sidelobe, Taylor amplitude, slot array antenna, millimeter-wave

## Abstract

To address the critical demand for high-gain, low-sidelobe, and high-efficiency antennas in W-band arrays, this work presents a low-sidelobe all-metal array antenna based on ridge gap waveguide technology. The design employs a three-layer contactless metal structure, integrating a stepped-ridge feeding network with Taylor amplitude distribution and a higher-order mode resonant cavity. This integration enables efficient power distribution and low-loss transmission while eliminating the need for conventional welding or bonding processes. Measurement results indicate that the antenna exhibits a reflection coefficient below −10 dB across the 92.5–103.5 GHz. The in-band gain exceeds 25.8 dBi with less than 1 dB fluctuation, and the radiation efficiency surpasses 78%. Specifically, the sidelobe levels in both E- and H-planes remain below −17.5 dB, reaching under −19.5 dB at 94 GHz, while cross-polarization is better than −30 dB. The proposed antenna demonstrates high gain, low sidelobe, and high efficiency, showing promising potential for applications in millimeter-wave radar, imaging, and 6G communication systems.

## 1. Introduction

The rapid development of mobile internet and 5G/6G communication technologies drives wireless systems to access broader spectrum resources in the millimeter-wave band. Against this backdrop, the W-band (75–110 GHz) has demonstrated significant application potential in high-capacity communications, high-resolution imaging, and automotive radar due to its atmospheric attenuation windows and substantial available bandwidth [1,2,3]. As a core component of these systems, the antenna’s performance directly determines the overall link budget and imaging quality. Therefore, the realization of array antennas with high gain, high efficiency, and stable radiation characteristics has become a major research focus.

However, W-band antenna design faces severe high-frequency challenges. Conventional high-gain antennas, such as horns [4,5,6] or parabolic reflectors [7,8,9], are bulky and difficult to integrate. While printed circuit board (PCB)-based microstrip patches [10,11,12] or substrate integrated waveguide (SIW) antennas [13,14] can achieve low profiles, they suffer from significant dielectric loss in the W-band, leading to sharply reduced radiation efficiency, and surface waves at radiating slots can degrade sidelobe levels. To address the loss issue, multilayer metal waveguide structures have been extensively investigated, such as slot arrays fabricated using diffusion bonding [15,16,17], yet these methods involve high cost and fabrication complexity. In recent years, gap waveguide technology, particularly ridge gap waveguide, has emerged as a promising approach for millimeter-wave antenna design, offering the distinct advantages of all-metal low-loss performance and non-contact assembly [18,19,20].

Although notable progress has been made in the study of gap waveguide-based array antennas, existing solutions still exhibit clear limitations in achieving a balanced combination of high-performance metrics. For instance, a design in [21] adopted an SIW resonant cavity and an RGW feeding network to achieve high aperture efficiency (>75%), but its sidelobe suppression (<−10 dB) remained moderate. Another study [22] utilized open-ended waveguide feeding to realize an ultra-wide bandwidth (35.7%), yet its number of structural layers and sidelobe performance (<−15 dB) leave room for improvement, and the complexity of the fully machined metal network is non-negligible. Notably, although a recent study [23] achieved high efficiency through a novel magnetoelectric dipole combined with a U-shaped T-junction, its sidelobe level (approximately −13 dB) remains relatively high. These studies demonstrate that, in the W-band, effectively achieving low sidelobe radiation without sacrificing gain, bandwidth, or efficiency remains a key and challenging technical problem.

To address this challenge, this work presents a fully metallic ridge gap waveguide slot array antenna incorporating Taylor-weighted feeding. The design introduces a stepped ridge feeding network based on Taylor amplitude distribution, which works in conjunction with a higher-order mode resonant cavity to achieve efficient, low-loss power distribution while significantly reducing the array sidelobe level. Employing a fully metallic three-layer non-contact structure, this approach eliminates dielectric losses and welding/bonding processes, ensuring high radiation efficiency at high frequencies and simplifying assembly. The fabricated 8 × 8 array antenna exhibits a sidelobe level better than −17.5 dB, a stable gain exceeding 26 dBi, and a radiation efficiency higher than 78% across the 92.5–103.5 GHz band, successfully achieving an outstanding overall balance between low sidelobe, high gain, and high efficiency.

## 2. Design Process

### 2.1. Antenna Element

Electromagnetic band-gap (EBG) structures are primarily categorized into two types: mushroom-type and pin-type configurations [24,25]. The pin-type EBG consists of periodically arranged metal pins enclosed between two parallel metal plates, with air gaps between the pins. This structure exhibits distinctly different propagation characteristics for electromagnetic waves of different polarizations.

When a TE-polarized wave (with the electric field parallel to the metal plates) impinges on the structure at an incident angle θ, the electric field cannot couple effectively with the metal pins, thus failing to excite guided-wave modes. In this case, the structure can be equivalently modeled as a dielectric substrate with perfect electric conductor (PEC) boundaries. Conversely, for TM-polarized waves (with the magnetic field parallel to the metal plates), significant currents are induced in the metal pins, exciting electromagnetic propagation dominated by a hybrid TEM-TM mode.

For TM modes propagating along the pin-type EBG, the wave vector $k_{p}$ must satisfy the transverse resonance constraint given by Equation (1) [26,27]. On the left-hand side of the equation, the total equivalent susceptance in the vertical direction is presented, which combines the reactance arising from the standing-wave oscillation inside the dielectric pins, ktan(kd), and the reactance contributed by the evanescent fields in the air gaps, $\alpha_{TM}tanh\left(\alpha_{TM}d\right)$. On the right-hand side, the vertical attenuation constant associated with the transverse wave propagation is given.

(1) $$\left(1-\eta \right)ktan\left(kd\right)-\eta \alpha_{TM}tanh\left(\alpha_{TM}d\right)=\alpha_{TM}$$

The expressions for the attenuation constant ($\alpha_{TM}$) and the coupling coefficient (η) are given by Equations (2) and (3), respectively:

(2) $$\alpha_{TM}=\sqrt{k_{p}^{2}-k^{2}}$$

(3) $$\eta =\frac{{k_{p}}^{2}}{{k_{p}}^{2}+{k_{t}}^{2}}$$

And $k_{t}$ can be expressed as Equation (4). p denoting the distance between the centers of adjacent pins and w representing the pin radius.

(4) $$k_{t}=\sqrt{\frac{2\pi }{p^{2}\left[ln\left(\frac{p}{2\pi w}+0.5275\right)\right]}}$$

Analysis shows that the propagation constant $k_{p}$ of the TM mode in the pin-type EBG structure depends on the pin height *d*. When the pin height lies within the range *λ*_0_/4 < *d* < *λ*_0_/2, the first TM mode is effectively suppressed, resulting in a stopband defined by 2*d* < *λ*_0_ < 4*d*.

Furthermore, the equivalent surface impedance of the pin-type EBG structure can be expressed by Equation (5):

(5) $$Z_{s}=j\sqrt{\mu_{0}/\varepsilon_{0}}\frac{1}{\varepsilon_{h}}tan\left(k_{h}d\right)$$

where $k_{h}=k_{0}\sqrt{\varepsilon_{h}}$. In this structure, *ε_h_* represents the relative permittivity of the medium surrounding the pins, and k_h_ is the propagation constant within that medium. When the pins are surrounded by air, *ε_h_* equals 1, and *λ*_0_ denotes the free-space wavelength. As *d* approaches *λ*_0_/4, *Z_s_* tends towards infinity, causing the pin-type EBG surface to exhibit high impedance characteristics. Consequently, electromagnetic waves cannot propagate along the surface in any direction.

**Figure 1:**
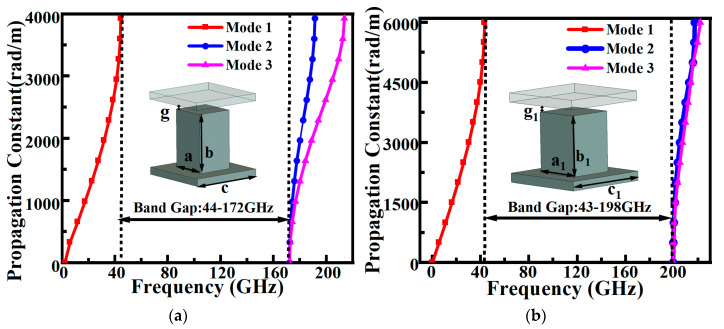
Dispersion diagram of pin-type EBG structure: (**a**) cavity layer pin, a = 0.4, b = 0.8, c = 0.8, g = 0.03; (**b**) feeding layer pin, a1 = 0.5, b1 = 0.55, c1 = 0.95, g_1_ = 0.03 (all dimensions are in mm).

To meet the requirements of different functional regions in the antenna, two pin units with distinct dimensions were optimized for the feeding network layer and the cavity layer in this work. The dispersion characteristics of these two EBG structures were analyzed using the Eigenmode Solver in CST Studio Suite. The simulated dispersion curves (Figure 1) indicate that the stopband of the feeding-layer pins spans from 43 GHz to 198 GHz, while the stopband of the cavity-layer pins covers 44 GHz to 172 GHz. The stopbands of both EBG structures fully encompass the W-band operating frequencies (75–110 GHz), ensuring effective suppression of parasitic modes and energy leakage across the entire operational band and providing a reliable foundation for the subsequent transmission line and cavity design.

**Figure 2:**
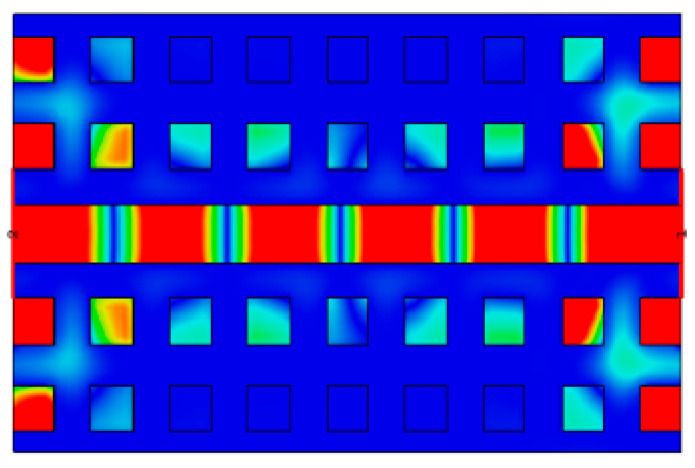
Electric field distribution.

**Figure 3:**
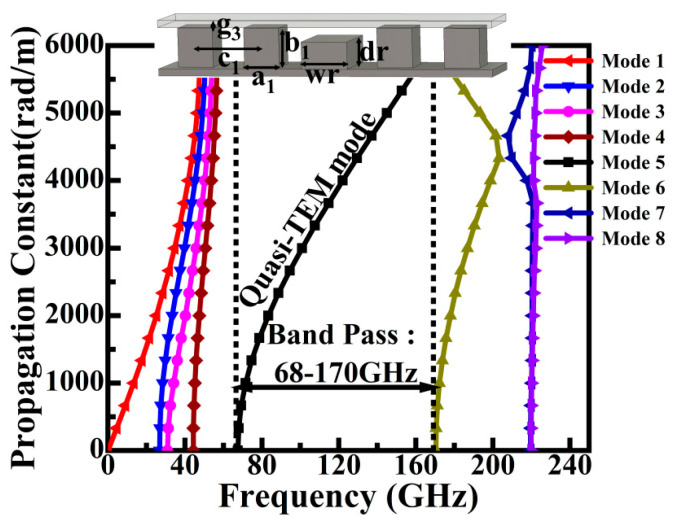
Structural diagram and dispersion relationship of ridge gap waveguide unit.

The ridge gap waveguide (RGW) serves as the key structure for achieving low-loss transmission. As shown in Figure 2, the electromagnetic waves propagate in the form of a quasi-TEM mode, confined within the low air gap above the metal ridge. Utilizing this air gap for propagation effectively eliminates the radiation and dielectric losses typically associated with conventional microstrip lines. The dispersion characteristics of the RGW unit cell were analyzed in this work (Figure 3), confirming that its passband spans 68–170 GHz and fully covers the W-band.

**Figure 4:**
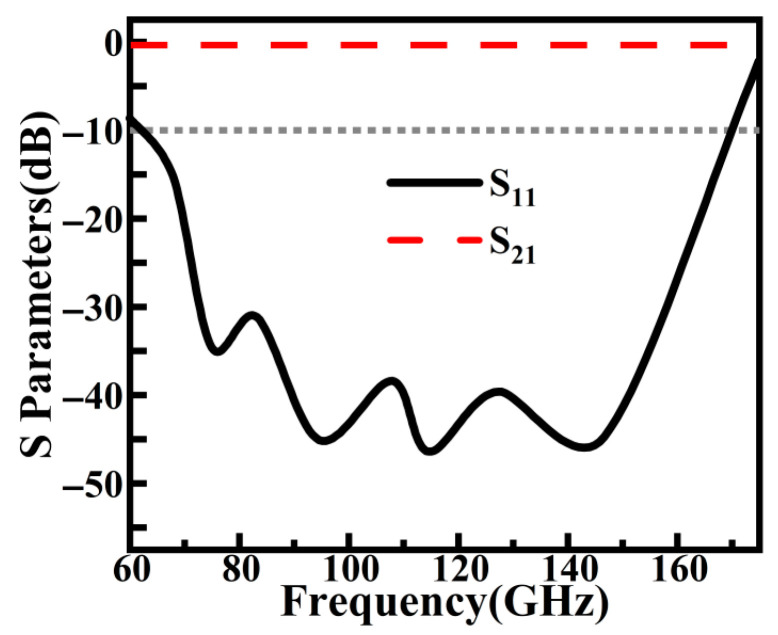
S_11_ and S_21_ of ridge gap waveguide.

To evaluate its transmission performance, the S-parameters of the RGW were simulated in this work. The results (Figure 4) show that over an extremely broad frequency range of 60–170 GHz, its return loss is better than 10 dB, and the insertion loss is very low. This demonstrates the excellent signal transmission capability of the RGW in the W-band, making it highly suitable as a feeding network for the array antenna.

**Figure 5:**
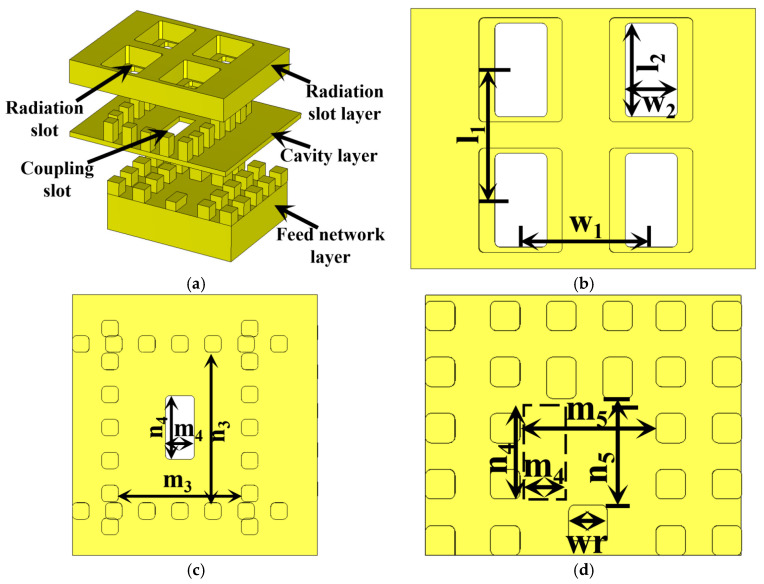
Structure of antenna element: (**a**) overall structure; (**b**) radiation layer, n_1_ = 2.5, n_2_ = 1.8, m_1_ = 2.5, m_2_ = 1; (**c**) cavity layer, n_3_ = 3.6, n_4_ = 1.55, m_3_ = 3, m_4_ = 0.7; (**d**) feeding layer, n_5_ = 1.8, m_5_ = 2.3 (all dimensions are in mm).

The antenna element serves as the core component for achieving efficient radiation. The adopted element design employs a three-layer structure comprising a radiating layer, a cavity layer, and a feeding layer (Figure 5). Following antenna array theory [27], the element spacing was chosen to keep the radiating slot separation below half of the free-space wavelength (λ_0_/2), thus suppressing grating lobes. In this design, the initial length of the radiating slots was *λ*_0_/2, and the initial distance of the coupling slots was *λg*/2. Precise optimization of the input impedance matching characteristics of the antenna element was achieved through parametric control of the radiating slot width.

**Figure 6:**
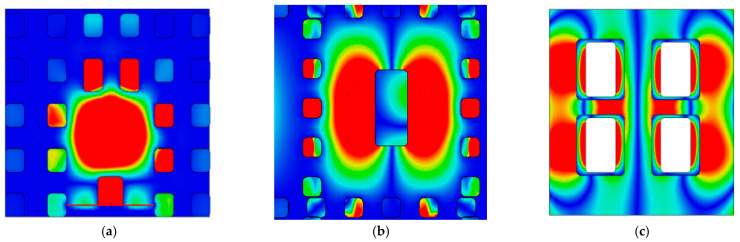
Electric field distribution diagram of antenna element: (**a**) feeding layer. (**b**) cavity layer. (**c**) radiation layer.

Figure 6 illustrates the electric field distribution within the three layers of the antenna unit. Clearly revealing the mechanism of electromagnetic energy transfer and conversion from the feeding network to the radiating slots. Energy is first coupled efficiently from the feeding layer to the upper higher-order mode resonant cavity through periodic coupling slots, exciting a TE_120_-mode standing-wave field distribution within the cavity. This mode further uniformly excites the four radiating slots arranged in a 2 × 2 configuration on the top layer, thereby forming a radiating subarray.

**Figure 7:**
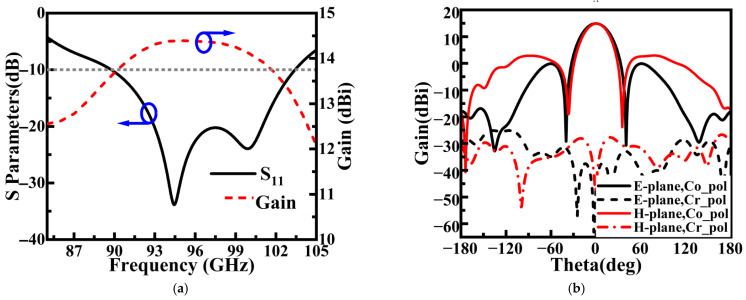
Simulation results of antenna element: (**a**) reflection coefficient and gain; (**b**) radiation pattern at 94 GHz.

A series of parametric analyses was conducted to characterize the element performance. As shown in Figure 7, the element achieves a return loss better than 10 dB over a 90–103 GHz bandwidth, corresponding to a relative bandwidth of 13.5%. At 94 GHz, the element gain reaches 14.4 dBi. The radiation patterns in both the E- and H-planes exhibit good symmetry, with half-power beamwidths narrower than 9.5° and cross-polarization levels below −25 dB. Notably, by optimizing the aperture field distribution, the first sidelobe levels in the H- and E-planes were suppressed to below −15 dB and −13 dB, respectively, providing a basis for achieving low sidelobe performance in the array.

### 2.2. Feeding Network

**Figure 8:**
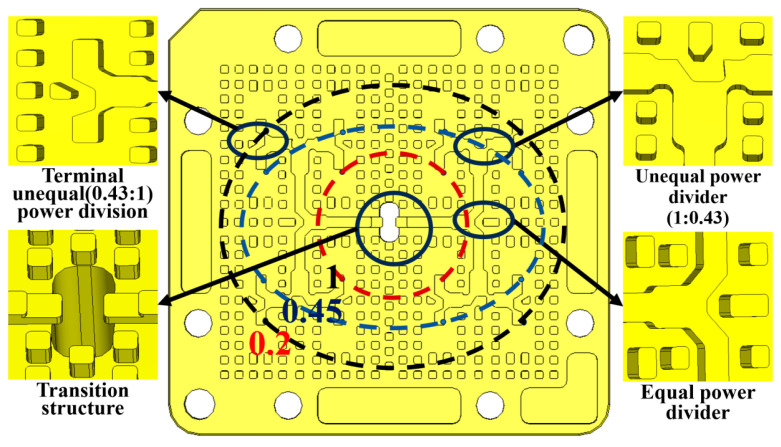
Unequal power distribution network.

In antenna array design, the amplitude distribution of the feeding elements directly determines the sidelobe characteristics of the radiation pattern. Taylor amplitude distribution [28,29] is commonly employed in engineering practice for sidelobe optimization. As shown in Figure 8, an unequal power-division network with one input and sixteen output channels (i.e., a 1:16 unequal power distribution network) is developed in this work based on a Taylor amplitude distribution.

**Figure 9:**
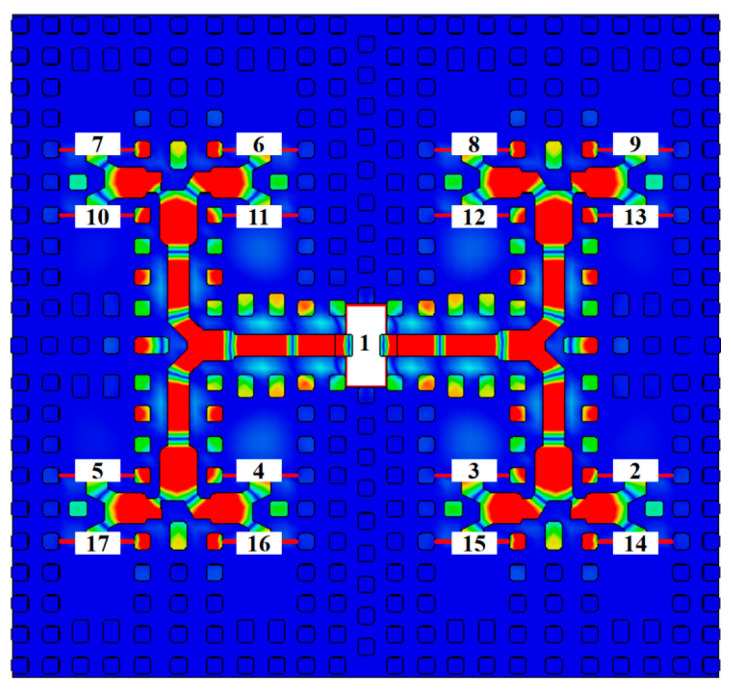
Unequal power distribution network electric field distribution.

To achieve array miniaturization, a mirror-symmetric layout was adopted for the feeding network. Although this layout introduces a 180° phase difference, the design compensated for this at all output ports (to nearly zero degrees) by incorporating a meticulously designed 180° phase-inversion compensation structure at the ends of the ridge waveguides, to ensure in-phase array excitation. The electric field distribution shown in Figure 9 demonstrates that the output amplitude distribution of the feeding network agrees well with the ideal Taylor amplitude distribution.

To realize precise unequal power division within a compact space, the network utilizes composite T-junctions. The critical power division is achieved through stepped ridges, where adjusting the width and length of the steps enables precise control over the power division ratio to meet the requirements of the Taylor distribution.

**Figure 10:**
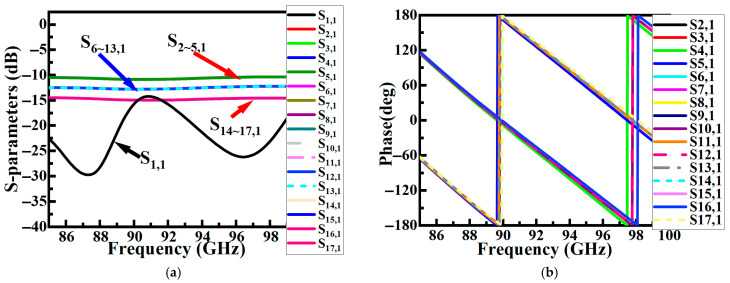
Simulation results of power distribution network: (**a**) reflection coefficient; (**b**) phase.

An isolated simulation of this 1:16 feeding network was performed. The results (Figure 10) indicate that across the 85–100 GHz band, the input port return loss is better than 13.5 dB with good phase consistency, thus validating the design of the feeding network.

### 2.3. Antenna Subarray

**Figure 11:**
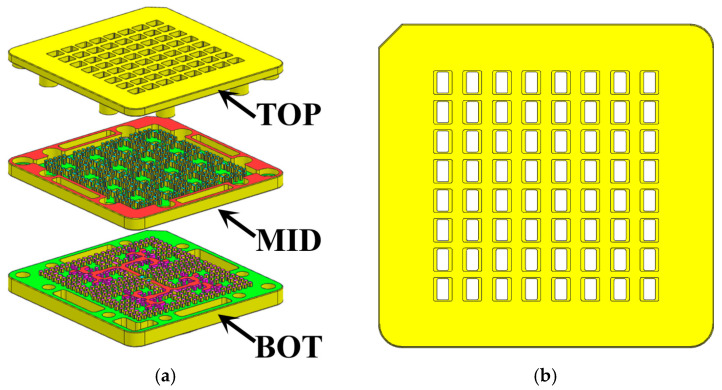

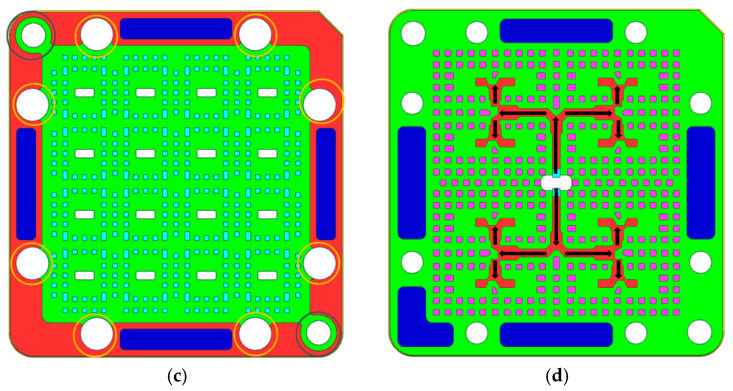
Antenna structure: (**a**) overall structure; (**b**) radiation layer; (**c**) cavity layer; (**d**) feeding layer.

**Figure 12:**
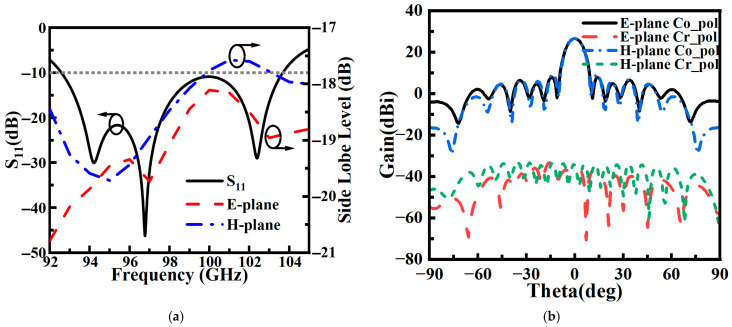
Antenna simulation results: (**a**) S_11_ and sidelobes; (**b**) E/H-plane pattern at 94 GHz.

The ridge waveguide feeding network, based on Taylor amplitude distribution, is integrated with the antenna elements to form a low-sidelobe array antenna, as illustrated in Figure 11. Figure 11b–d show front views of the radiation layer, cavity layer, and feed layer, respectively. In Figure 11d, arrows are used to indicate the propagation path of the electromagnetic signal. Simulation results (Figure 12) show that the antenna exhibits an S11 below −10 dB across the 92.5–103.5 GHz frequency band, with both E- and H-plane sidelobe levels remaining below −17.5 dB. At the 94 GHz atmospheric window frequency, the radiation pattern demonstrates excellent symmetry: the measured 3 dB beamwidth is below 8.9° in both principal planes, with sidelobe levels reaching −19.8 dB (E-plane) and −19.5 dB (H-plane), while cross-polarization suppression exceeds −30 dB, indicating good polarization purity.

## 3. Experimental Results

**Figure 13:**
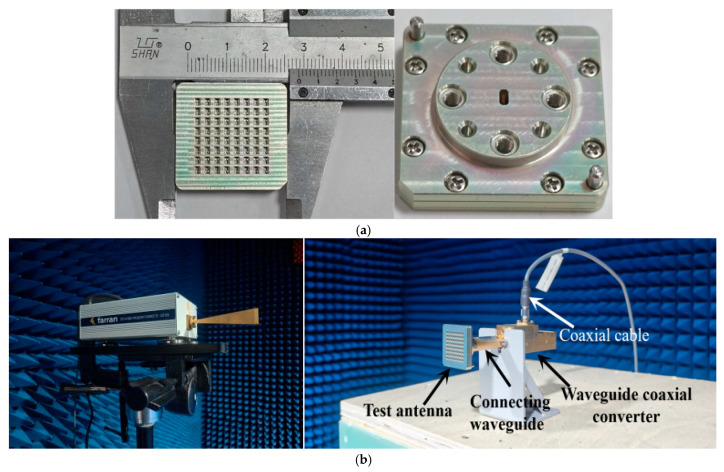
Photographs of (**a**) fabricated prototype and (**b**) measurement setup.

To validate the design, the proposed 8 × 8 ridge gap waveguide array antenna was fabricated and tested in this work. The antenna prototype was fabricated as a single piece using computer numerical control (CNC) milling technology, with all metal layers made of aluminum to balance weight and conductivity. The overall dimensions of the final fabricated antenna prototype are 28.4 mm × 27.4 mm × 6.97 mm, with its photograph and testing setup shown in Figure 13.

**Figure 14:**
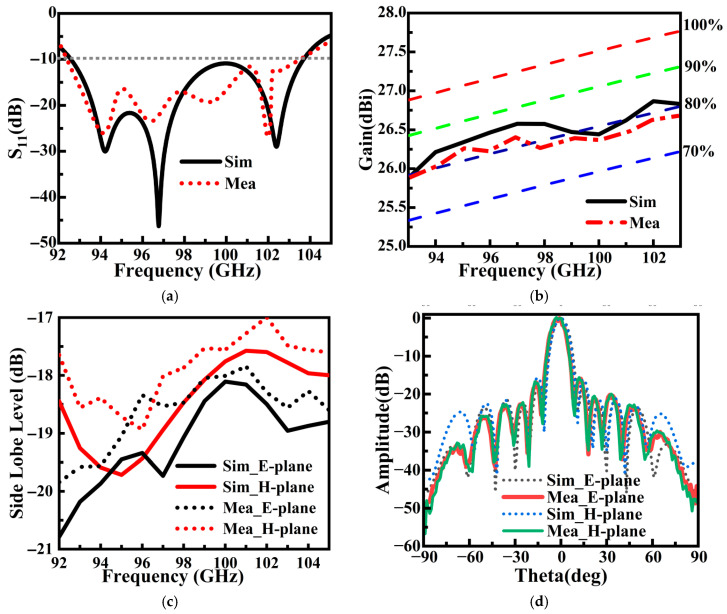
Comparison of simulation and measurement results: (**a**) S11; (**b**) gain and efficiency; (**c**) sidelobes; (**d**) E/H-plane pattern at 94 GHz.

To ensure accuracy and reliability, a measurement strategy involving averaging over multiple repeated batches was employed. Comparative analysis of the measured and simulated data, shown in Figure 14, indicates a high degree of consistency in the antenna’s core performance metrics. Figure 14a,b demonstrate that the antenna exhibits a reflection coefficient better than −10 dB across the 92.5 GHz to 103.5 GHz frequency band, with the measured results largely agreeing with the simulation trend. Over the entire operating band, the measured gain remains above 25.8 dBi, and the efficiency exceeds 78%. Figure 14c shows that the sidelobes in both the E- and H-planes are effectively suppressed below −17.5 dB. The radiation patterns in the E- and H-planes at the typical frequency of 94 GHz are presented in Figure 14d, where the first sidelobe levels are as low as −19.8 dB and −19.5 dB, respectively. Although slight sidelobe elevation and local pattern fluctuations were observed, the overall radiation characteristics maintain an engineering-acceptable level of agreement. Discrepancies mainly arise from fabrication constraints, including micron-level dimensional tolerances of metal parts, ohmic losses due to surface roughness, and sub-wavelength alignment errors during the multilayer assembly process.

Table 1 summarizes the performance of the proposed antenna array, systematically validating the comprehensive advantages of the presented ridge gap waveguide array design. Compared with existing millimeter-wave arrays, this work achieves an excellent balance across multiple key performance indicators, including low sidelobe levels, high gain, high radiation efficiency, and compact size. Furthermore, the fully metallic gap waveguide fabrication process demonstrates enhanced manufacturability and assembly simplicity, offering lower cost and higher precision compared to conventional methods. The performance exhibits distinct competitiveness among related works.

## 4. Conclusions

To address the comprehensive requirements for high gain, high efficiency, and low sidelobes in the millimeter-wave band, a low-sidelobe fully metallic ridge gap waveguide array antenna for W-band applications was designed, fabricated, and tested in this work. The antenna integrates a stepped-ridge feeding network based on Taylor amplitude distribution with a higher-order mode resonant cavity within a three-layer fully metallic non-contact structure. Measurement results demonstrate that the antenna achieves good impedance matching across a 92.5–103.5 GHz bandwidth, with an in-band gain exceeding 25.8 dBi, gain fluctuation below 1 dB, and radiation efficiency higher than 78%. In particular, the sidelobe levels in both E- and H-planes are suppressed below −17.5 dB, further reaching below −19.5 dB at 94 GHz, while the cross-polarization level remains better than −30 dB. These performance metrics fully validate the effectiveness of the synergistic design approach combining the fully metallic ridge gap waveguide structure and the Taylor-weighted feeding scheme adopted in this work. The design successfully achieves a favorable balance between high radiation efficiency and low sidelobe levels, providing an effective solution to the common performance trade-offs in high-performance W-band array antenna design.

## Figures and Tables

**Table 1:** Performance comparison between related antenna arrays.

Ref.	[21] 2022	[22] 2022	[2] 2024	[14] 2023	[23] 2024	This Work
Structural	SIW Cavity + RGW Feed	Open-ended Waveguide-fed Slot + Hybrid Feed	SIW Slot Array + EBG for Leakage Suppression	Microstrip Patch + Multi-layer Parasitic Patches	Side-connected ME Dipole + RGW Feed	RGW Taylor-feed Network + Cavity-coupled Slot Array
Fabrication	Mixed PCB & Machining	Machining (CNC)	Multilayer PCB	HDI Process	PCB + Machining	Full-metal Machining
Array	8 × 8	8 × 8	8 × 16	8 × 8	8 × 8	8 × 8
Aperture (mm)	26.0 × 34.0 × 11.4	32 × 32 × 4	32 × 18 × 1.413	15.2 × 15.2 × 0.5699	19.2 × 19.2 × 2.72	28.4 × 27.4 × 6.97
BW (GHz)	97.8–107	78–110	91.2–96.7	85.2–110	81.5–108.6	92.5–103.5
SLL (dB)	<−10	<−15	<−15	-	−13	<−17.5
Gain (dBi)	24–26.5	24.3–26.8	23–24.5	21–22.2	22.5–26.6	25.8–26.3
Eff (%)	>75	>70	>49.1	71.92	>60	>78
XPD (dB)	<−20	<−32	<−50	<−25	<−30	<−30

## Data Availability

All data are available from the author.
